# A study examining the correlation between serum 25-OH-VitD levels, CD3-CD19+ B lymphocytes, and the risk of early spontaneous abortion in pregnant women

**DOI:** 10.1097/MD.0000000000034338

**Published:** 2023-07-14

**Authors:** Cuifang Ai

**Affiliations:** a Department of Clinical Laboratory, Hubei Maternity and Childcare Hospital, Hubei Province Women and Children Hospital, Wuhan, China.

**Keywords:** ESA, 25-OH-VitD, CD3-CD19+ B lymphocytes, risk factor

## Abstract

The objective of this retrospective cohort study was to investigate the correlation between serum 25-OH-VitD levels, coagulation function, immune factors, and the risk of spontaneous abortion in the first trimester of pregnancy. Additionally, independent risk factors for spontaneous abortion in the first trimester of pregnancy were identified. A total of 412 pregnant women who attended Hubei Maternal and Child Health Care Hospital between October 2021 and February 2022 were included in the study. Of these, 221 met the eligibility criteria and were categorized into the early spontaneous abortion case group (n = 107) or the normal pregnancy control group (n = 114). The serum levels of 25-OH-VitD, CD3 + CD19- T lymphocytes, CD3-CD19 + B lymphocytes, NK (Natural Killer) cells, TNF-α (tumor necrosis factor-α) and coagulation factors (D-dimer, Protein C, Protein S) in both these groups were measured during early pregnancy (within 12 weeks) and evaluated using logistic regression analysis. Compared to the control group, body mass index, Protein S, CD19 + CD3-B lymphocytes, and 25-OH-VitD were significantly lower in the spontaneous abortion group during early pregnancy (*P* = .001; *P* = .004; *P* = .009; *P* = .001), blood glucose (fasting) and TNF-α significantly increased (*P* = .001; *P* = .046). Logistic regression analysis of potential mixed factors showed that fasting blood glucose and TNF-α were significantly different from the control group and were positively correlated (*P* = .001, *P* = .038). Fasting blood glucose 0R value is 2.264, 95% confidence interval is 0.043~0.25, TNF-α 0R value is 0.126, 95% confidence interval is 0.800~0.972. CD19 + CD3-B cells and 25-OH-VitD were correlated with spontaneous abortion (*P* = .005; *P* = .001), respectively 0R value and 95% confidence interval being −0.007 (1.002~1.012), −0.179 (1.139~1.256). Risk factors for spontaneous abortion in the first trimester (<12 weeks) of pregnancy include fasting glucose tolerance, decreased CD19 + CD3-energy B lymphocytes and 25-OH-VitD, and abnormal increase of TNF-α. Therefore, it is recommended that women with fertility needs be examined as early as possible to avoid adverse outcomes.

## 1. Introduction

Early spontaneous abortion (ESA) is one of the most common pregnancy complications, with over 80% occurring before the 12th week of pregnancy. Globally, pregnant women have an incidence rate of around 15%, while in China, the incidence of ESA is approximately 10% to 14%.^[1–3]^ The pathogenesis of ESA is complex and can be attributed to various causes, including congenital genetic factors such as fetal chromosome abnormalities, acquired factors like maternal endocrine and metabolic disorders, uterine dysplasia or malformation, immune dysfunction, and reproductive tract infections, among others. However, currently, the causes of 50% of all ESA cases remain unclear.^[4]^ In recent years, scholars have increasingly focused on identifying the risk factors of ESA in pregnant women, and identifying early risk indicators has become a critical area of research. In this context, our study analyzed the serum levels of 25-OH-VitD, immune cells (CD3 + CD19-T lymphocytes, CD3-CD19 + B lymphocytes, NK cells), tumor necrosis factor (TNF-α), and coagulation function (D-dimer, Protein C, Protein S) in both normal pregnant women and those with ESA during early pregnancy. This analysis aimed to explore the independent risk factors of ESA, thereby providing a theoretical and research basis for the prevention and treatment of this disease.

## 2. Materials and methods

### 2.1. General information

**Figure 1.**
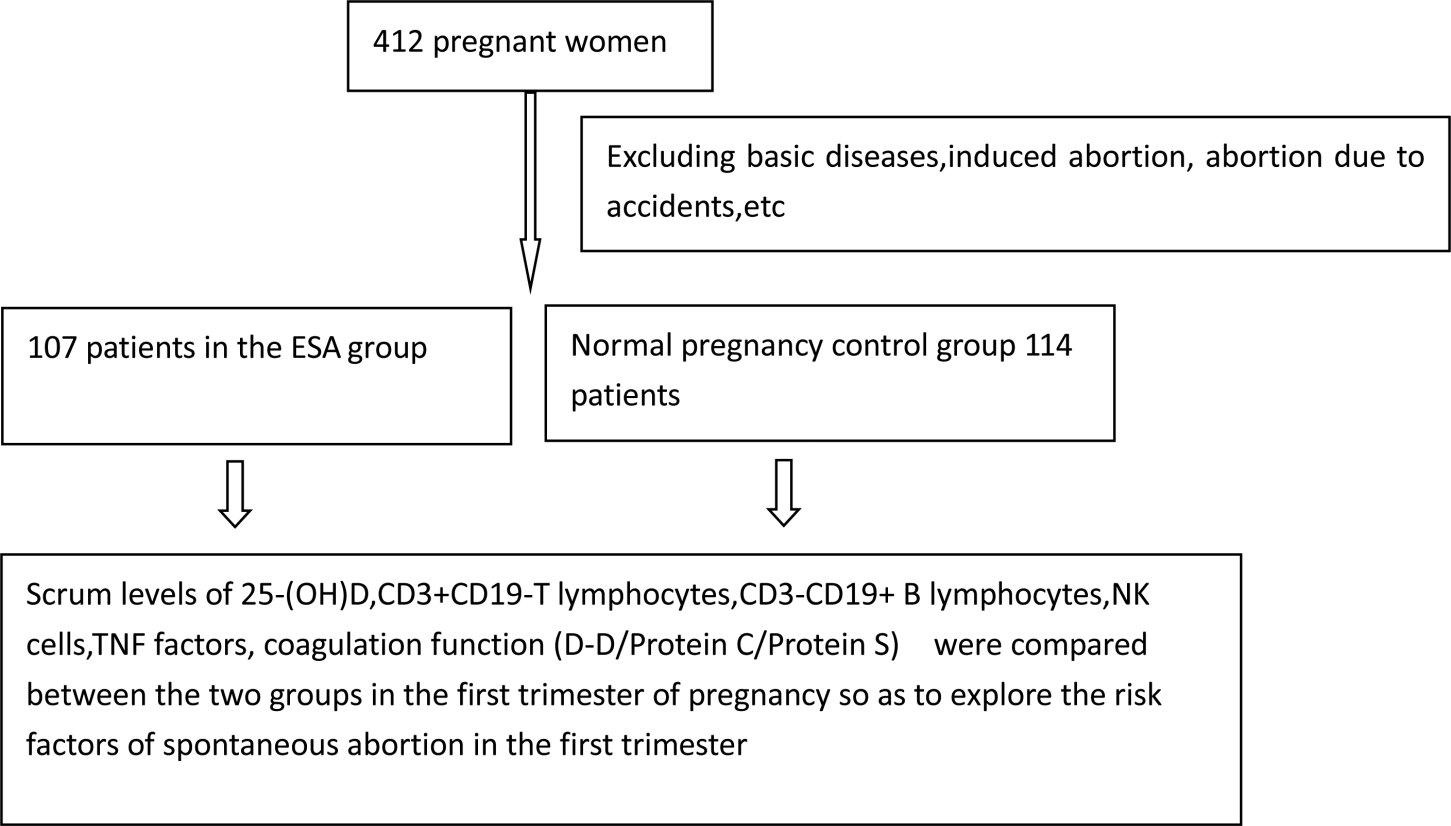
Flowchart depicting the study population. Research process like this, 412 pregnant women, excluded with chronic conditions such as hypertension, diabetes, kidney disease, liver disease, induced abortion, and accidental abortion before pregnancy.

This retrospective cohort study gathered data from 412 pregnant women who received medical treatment at the Hubei Maternal and Child Health Hospital from October 2021 to February 2022.The study population were shown in Figure 1, excluded women with chronic conditions such as hypertension, diabetes, kidney disease, liver disease, induced abortion, and accidental abortion before pregnancy. The patients were then divided into 2 groups based on whether they had an abortion within 12 weeks of pregnancy: the control group (114 cases) and the abortion group (107 cases).

### 2.2. Inclusion criteria

Inclusion criteria:

1. No serious systemic diseases.
2. Meets the diagnostic criteria for ESA as outlined in the 9th edition of Obstetrics and Gynecology.
3. Must have undergone prenatal tests including fasting blood glucose, serum 25-OH-VitD, CD3 + CD19-T cells, CD3-CD19 + B cells, NK cells, TNF-α factors, homocysteine, and tests for coagulation functions (D-dimer, Protein C, Protein S), among others.
4. The study was approved by the Ethics Committee of the hospital. All patients were informed about the process of the case collection and their consent was obtained. The normal pregnancy control group comprises of women who gave birth after 28 weeks of gestation.

## 3. Methods

### 3.1. Basic data collection

Basic information including age, height, weight, gestational age, and reproductive history.

### 3.2. Detection method

Fasting peripheral elbow venous blood was collected from pregnant women during pregnancy confirmation, centrifuged at 3000 r/minute and 10 minute, and the upper serum (D-dimer, protein C, and protein S were used to obtain anticoagulant plasma by the same method). The specific detection procedures followed the reagent instructions. Details of the detection index methods and principles were shown in Table 1.

**Table 1:** Test items and method phase information table.

Test item	Detection method	Reagent	Company
Glu (fasting)	Glucose oxidase	Glucose assay kit	Abbott
25-OH-VitD	Colloidal gold process	25-OH-VitD test kit	Primed
CD3^+^CD19^-^T lymphocyte	Flow fluorimetry	Lymphocyte subsets test kit	BD
CD3^-^CD19^+^B lymphocyte			
CD16^+^CD56^+^NK cells			
TNF-α	ELISA	TNF-α detection kit	Shanghai Kanglang
D-dimer	immunoturbidimetry	D-dimer assay kit	Instrumentation LaboratoryCo.
Protein C	Substrate chromogenesis	Protein C activity assay kit	Siemens Health care Diagnostics
Protein S	Solidification	Protein S activity assay kit	Products GmbH
Hcy	Circulase	Homocysteine assay kit	Abbott
ANA	Indirect immunofluorescence	Anti-nuclear antibody test kit	AESKU

AESKU = AESKU Corporation, Germany; ANA = Anti-nuclear antibody; BD = Becton, Dickinson and Company; Hcy = homocysteine; TNF-α = tumor necrosis factor-α.

### 3.3. Statistical

Data were processed using SPSS24.0 statistical software. Measurement data were expressed by χ̅±s, and *t* test was performed. χ^2^ test was used for statistical data. Logistic regression analysis was used to examine the influencing factors of spontaneous abortion. *P* < .05 was considered statistically significant.

## 4. Results

### 4.1. General situation comparison

There were no significant difference in the mean age between the 2 groups (*P* > .05). However, there were notable distinctions in terms of the number of pregnancies and body mass index (BMI). The outcomes revealed that the spontaneous abortion group had significantly more pregnancies than the normal group (*P* < .001) and a significantly higher BMI (*P* = .001), as demonstrated in Table 2.

**Table 2:** Characteristics of women in different groups.

Basic characteristics	Normal control group (X̅ ± s)	Case group (X̅ ± s)	*P*
Age (yr)	31.99 ± 4.86	31.47 ± 3.84	.385
BMI (kg/m^2^)	23.32 ± 3.57	24.84 ± 2.80	.001
Number of pregnancies	1.60 ± 069	2.42 ± 1.13	<.001
Single pregnancy	57 (50.00%)	19 (17.75%)	
Multiple pregnancy	57 (50.00%)	88 (82.24%)	

BMI = body mass index.

Based on the data presented in Table 3, the peak fertility age among the women in this case study group was between 25 and 30 years, with the majority of women having had 2 pregnancies. However, the occurrence of recurrent abortion (≥3 times) was more prevalent among women aged 30 to 35 years. This increase in the number of abortions resulted in a higher number of pregnancies when compared to other age groups, which is consistent with literature reports. The pregnancy-related laboratory indicators of the 2 groups were compared, and the data showed normal distribution. The statistical analysis indicated that compared to the normal group, women in the case group had significantly lower levels of Protein S, CD19 + CD3- B lymphocytes, and 25-OH-VitD, with statistical significance (*P* = .001; *P* = .004; *P* = .009; *P* = .001). Additionally, their blood glucose (fasting) and TNF-α levels were significantly higher (*P* = .001; *P* = .046). Refer to Table 4 for detailed information regarding these results.

**Table 3:** Statistical of age, pregnancy and abortion of women in the case group.

Age (yr)	The number of pregnancies			The number of abortions (times)		
	1	2	≧3	1	2	≧3
20~25	0	3 (2.80%)	0	0
3 (2.80%)	0	25~30	11 (10.28%)	24 (22.43%)
11 (10.28%)	27 (25.23%)	14 (13.08%)	7 (6.54%)	30~35
6 (5.60%)	15 (14.02%)	19 (17.76%)	19 (17.76%)	11 (10.28%)
10 (9.35%)	35~40	2 (1.87%)	7 (6.54%)	7 (6.54%)
4 (3.74%)	7 (6.54%)	4 (3.73%)	≧40	0 ()
1 (0.93%)	0	1 (0.93%)	0	0

**Table 4:** Changes of relevant test indexes in different groups.

Inspection index	Normal control group (X̅ ± s)	Case group (X̅ ± s)	*P*
Glu (fasting)(mmol/L)	5.83 ± 0.55	5.90 ± 0.69	<.001
Protein C (%)	91.59 ± 22.53	103.37 ± 14.88	.627
Protein S (%)	80.79 ± 18.56	72.41 ± 23.82	.004
D-dimer (µg/mL)	0.389 ± 0.28	1.04 ± 4.36	.337
Hcy (µmol/L)	5.83 ± 1.43	6.36 ± 1.57	.085
TNF-α (pg/mL)	7.81 ± 5.25	10.67 ± 7.48	.046
ANA*	24	79	.859
Negative	21 (87.50%)	68 (86.07)	
Positive	3 (12.50%)	11 (13.90%)	
CD3 + CD19-T (number/µL)	1460.86 ± 367.7	1283.19 ± 517.30	.563
CD19 + CD3-B (number/µL)	306.82 ± 132.11	213.89 ± 99.87	.009
CD4/CD8	1.53 ± 0.54	1.55 ± 1.20	.382
CD16 + CD56 + NK (number/µL)	355.83 ± 191.11	287.49 ± 191.73	.274
25-OH-VitD (µg/mL)	34.43 ± 11.58	17.31 ± 9.53	<.001

TNF-α = tumor necrosis factor-α.

* ANA unit of comparison is number of people.

### 4.2. Correlation between these risk factors and ESA

In this study, logistic regression analysis was conducted on potential mixed factors such as fasting blood glucose, Protein S, CD19 + CD3-B lymphocytes, 25-OH-VitD, and TNF-α. The results showed that the ESA group had significant differences in fasting blood glucose compared to the control group, with a positive correlation (*P* = .001), 0R value of 2.264, and a 95% confidence interval of (0.043~0.25). Additionally, TNF-α had a 0R value of 0.126 and a 95% confidence interval of (0.800~0.972), with a positive correlation (*P* = .038). On the other hand, CD19 + CD3-B lymphocytes and 25-OH-VitD were negatively correlated with spontaneous abortion (*P* = .005; *P* = .001), with 0R values of −0.007 (95% confidence interval: 1.002~1.012) and −0.179 (95% confidence interval: 1.139~1.256), respectively, Please refer to Table 5 for further details.

**Table 5:** Correlation between blood glucose (fasting), protein S, CD19^+^CD3^-^, 25-OH-VitD levels and ESA, odds ratio (ORs) and 95% confidence interval (CI) in pregnant women.

Project	ESA	
	ORs (95%CI)	*P*
Glu (fasting)	−2.264 (0.043~0.25)	<.001
Protein S	0.018 (1.000~1.036)	.056
CD19^+^CD3^-^B	−0.007 (1.002~1.012)	.005
25-OH-VitD	−0.179 (1.139~1.256)	<.001
TNF-α	0.126 (0.800~0.972)	.038

ESA = early spontaneous abortion, TNF-α = tumor necrosis factor-α.

## 5. Discussion

The incidence of spontaneous abortion has increased in recent years due to delayed childbirth and increased work pressure among women. Early pregnancy, before 12 weeks, is the most common period for ESA, as the embryo has not yet fully developed. The causes of ESA include maternal factors, immune dysfunction, embryonic factors. Despite numerous studies on the etiology, the specific causes and pathogenesis of ESA have yet to be fully understood. As a result, preventing related risk factors has become a crucial focus of clinical inquiry.

In this study, the risk of spontaneous abortion during the first trimester was found to be higher in women with BMI outside the normal range, consistent with previous literature. Sugiyama T^[5]^ noted that common risk factors for progressing from normal pregnancy to complete abortion in the first 3 months included increasing maternal age and high pre-pregnancy BMI. While guidelines for optimal weight gain during pregnancy remain contentious, Goldstein RF^[6]^ found that lower or higher gestational weight gain within the BMI range was associated with a greater risk of adverse maternal and fetal outcomes. Therefore, controlling weight in early pregnancy is crucial in preventing ESA. The 2020 edition of the Chinese Expert Consensus on the Diagnosis and Treatment of Spontaneous Abortion^[7,8]^ suggests that patients with one history of abortion have a lower risk in subsequent pregnancies and higher success rate. The fertility peak for women in this study group was between 25 and 30 years old, with most having 2 pregnancies. Women with more than 3 abortions had a significantly higher risk of recurrent abortion, with up to 80% of such cases resulting in recurrent abortion. Among the 37 women in the case group who had been pregnant more than 3 times, 21 experienced ESA, accounting for 70%, which aligns with the expert consensus.

Optimizing blood glucose control during pregnancy can help lower the risk of diabetes. However, many women are unaware of abnormal blood glucose levels until their first prenatal checkup. The mean fasting blood glucose levels were 5.83 ± 0.55 and 5.90 ± 0.69 in the 2 groups examined in this study. The average level in the case group met the diagnostic criteria for impaired fasting glucose tolerance (IFG) according to the 2017 edition of “Diabetes Medical Care Standards,” which states that pregnant women with fasting glucose levels of 5.9 to 6.9 mmol/L should receive this diagnosis.^[9]^ A cohort study conducted by Wei Y et al^[10]^ on 6447,339 women aged 20 to 49 in China found that early pregnancy with IFG increased the risk of adverse pregnancy outcomes, including spontaneous abortion, macrosteria, small for gestational age, and perinatal infant death. In this study, a significant correlation was observed between fasting blood glucose levels and ESA in the case group (*P* = .001). Another study conducted by Zolghadri et al,^[11]^ which examined the incidence of abnormal glucose tolerance in patients with spontaneous abortion, found that patients with a history of repeated abortion had a higher rate of impaired glucose tolerance than the normal group. In our study, the mean fasting blood glucose levels in the ESA population were significantly higher than those in the control population, supporting the findings of the studies mentioned earlier.

Currently, studies suggest that coagulation disorders may contribute to first-trimester abortion.^[12]^ Pregnancy is already a hypercoagulable state due to the lack of antithrombotic factors, which can cause placental thrombosis resulting in fetal death and subsequent loss.^[13]^ It has also been found that an abnormal coagulation-fibrinolytic system is closely related to spontaneous abortion, often leading to thrombosis of uterine tissue and placenta, resulting in fetal ischemia and eventually abortion.^[13–15]^ Components of the body natural anticoagulant system, such as Protein S and Protein C, can reflect the body coagulation function. Deficient women have a higher risk of forming blood clots in the placenta, causing placental insufficiency, microthrombi formation in uterine spiral arteries or villus vessels, or even multiple placental infarction, resulting in poor perfusion of utero-placental circulation and an increased risk of fetal death and abortion.^[12]^

Currently, more studies believe that lower Protein S is closely related to recurrent abortion, but it is uncertain whether it is significant in early pregnancy spontaneous abortion. Mukhtar B^[16]^ compared Protein C and Protein S levels in pregnant women with recurrent abortion in the first and second trimesters of pregnancy and women with normal pregnancy for over a year, and found that Protein S deficiency was more common than Protein C in the first trimester. Protein S can enhance the anticoagulant effect of Protein C and retrograde bind various coagulation factors, inhibiting the activation of coagulation factors and regulating the coagulation process.^[16]^ In this study, Protein C and Protein S levels were compared between 2 groups, and it was found that the Protein S level of patients in the ESA group was significantly lower (72.41 ± 23.82, *P* = .004), consistent with Mukhtar B study. However, correlation analysis showed no direct correlation between the 2 groups (*P* = .056), and it cannot be regarded as an independent risk factor. Further studies with larger sample sizes are needed in the later stages.

In addition to maintaining the balance of calcium and phosphorus metabolism in the human body and participating in the regulation process of transcription of some proteins, 25-(OH)D plays a vital role in the reproductive system. This role may be related to a series of adverse pregnancy outcomes, such as spontaneous abortion, preeclampsia, and gestational diabetes.^[1]^ Several current studies suggest that preterm birth is associated with vitamin D deficiency during pregnancy. However, the association between vitamin D deficiency in the first trimester of pregnancy and abortion is not clear.^[17]^ Some experts believe that a variety of enzymes synthesized in the human placenta and decidua are involved in the vitamin D metabolic pathway. Consequently, low vitamin D levels are considered a risk factor for early abortion.^[18]^ In a study of pregnant women in Iraq, Kasim SF^[19]^ found that 95% of spontaneous abortion women had vitamin D deficiency, while only 17.5% of pregnant women did. Therefore, he believes that adequate vitamin D can promote placental blood vessel and fetal growth, enhance immune cell function, and help prevent pregnancy complications and abortion. This study also found that the vitamin D level of pregnant women in the normal group was significantly higher than that in the ESA group. Statistical analysis showed that the serum 25-(OH)D level was negatively correlated with the occurrence of ESA. This suggests that decreasing serum 25-(OH)D levels during pregnancy may increase the risk of ESA. Further exploration into the relevant mechanism is needed in later basic trials.

Vitamin D, it can inhibit the continuous proliferation of activated B lymphocytes and induce apoptosis to regulate the production of autoantibodies. Vitamin D deficiency leads to the over-activation of B lymphocytes, resulting in excessive production of autoantibodies and abnormal immune function,^[20]^ as discovered by Ota K et al^[21]^ They found that the level of peripheral CD19 + CD3-B lymphocytes in patients with vitamin D deficiency was significantly higher than that in the normal population. He believed that the elevated CD19 + CD3-B lymphocytes manifested autoimmune activation, which would inhibit the growth of placental trophoblast cells, induce fetal-maternal interface thrombosis, inflammation, and destroy maternal immune tolerance leading to abortion. It is also possible that an abnormally high level of B lymphocytes produces numerous autoantibodies, which disturbs the autoimmune system, leading to autoantibodies attacking the embryo, resulting in miscarriage. Medical professionals debate the level of B lymphocytes leading to abortion, and experts believe that the reduction of peripheral CD19 + CD3-B lymphocytes may cause spontaneous abortion.^[22]^ Slawek A used flow cytometry to detect the B lymphocyte count and the expression levels of cytokines IL-35 and IL-10 in peripheral blood, uterine draining lymph nodes, uterus, and deciduae of mice in the early stages of pregnancy. The study found that the number of local and peripheral B cells and the secretion of IL-35 reduced in mice prone to abortion in early pregnancy, negatively affecting normal pregnancy. A retrospective study discovered that the number of peripheral CD19 + CD3-B lymphocytes was significantly lower in women who miscarried than in those who had normal pregnancies,^[23]^ consistent with the study. Comparison of the 2 groups found that the lymphocyte levels were significantly lower in the abortion group than in the normal group (*P* = .009). Correlation analysis between this index and abortion indicated a significant negative correlation. The OR value and 95% confidence interval were −0.007 (1.002~1.012) respectively. This can be used as an independent risk factor for ESA occurrence, which may be caused by the deficiency of certain protective cytokines because of the decrease of B lymphocytes, leading to adverse pregnancy outcomes. Follow-up studies will explore related mechanisms further.

Furthermore, uterine environmental inflammation such as immune dysfunction in pregnant women and long-term high exposure to TNF-α may also play an important role in its etiology.^[24]^ Studies have shown that successful pregnancy requires the participation of inflammatory response, and TNF-α is considered to be a key cytokine that plays a crucial role in the establishment and maintenance of pregnancy. Excessive TNF-α is associated with adverse pregnancy outcomes.^[25]^ Wen Y^[1]^ [Bibr R8][Bibr R25]conducted a basic study by collecting decidua tissues of ESA patients and normal pregnancy population and found that the level of TNF-α in decidua tissues of ESA group increased significantly. He suggested that long-term stimulation with high TNF-α levels could induce abnormal activation of TNFR/NF-κB/FTH1 signaling, leading to excessive apoptosis of decidua, unstable implantation and ESA. However, our study group also showed that there was a significant difference in TNF-α between normal pregnancy and ESA group at the beginning of pregnancy (*P* = .046), that is, the average level of TNF-α in patients with ESA was significantly increased, and was correlated with the occurrence of abortion, with a 0R value of 0.126, 95% confidence interval (0.800–0.972). This is consistent with relevant research results.

## 6. Conclusion

In conclusion, IFG, low levels of CD19 + CD3- energy B lymphocytes, peripheral blood vitamin D, and an abnormal rise of TNF-α in pregnant women at the beginning of pregnancy, particularly before 12 weeks, increase the risk of ESA. However, follow-up studies need to confirm these findings through large-sample, multi-center studies combined with mechanism studies. Clinicians can also integrate routine early pregnancy screening and early diagnosis intervention to prevent adverse pregnancy outcomes.

## 7. Limitations and outlook

It is important to note this was a retrospective case analysis with a limited sample size that was conducted at a single center. Additionally, most women do not typically take vitamin D supplements until their pregnancy is confirmed. This may have confounded the investigation of oral vitamin D supplementation as a factor in ESA.

Future research should focus on abnormal test results at different gestational times, as well as on oral drug supplementation, clinical treatment, pathogenesis, and other related areas. In addition, multi-center studies are needed to expand the sample size and thus overcome one of the limitations of this study. Moreover, further prospective studies are required to explore underlying mechanisms and to validate the present results. By comprehensively and systematically analyzing the etiology of ESA, better preventive measures can be implemented in clinical practice.

## Acknowledgments

I would like to acknowledge the Director of our clinical laboratory Jianbo Xia for inspiring my interest in the development of innovative technologies.

## Author contributions

**Conceptualization:** Cuifang Ai.

**Data curation:** Cuifang Ai.

**Methodology:** Cuifang Ai.

**Supervision:** Cuifang Ai.

**Writing – original draft:** Cuifang Ai.

**Writing – review & editing:** Cuifang Ai.
